# Supplementary material for: The effects of cholesterol accumulation on Achilles tendon biomechanics: A cross-sectional study
Source: PLoS One. 2021 Sep 16;16(9):e0257269. doi: 10.1371/journal.pone.0257269 (PMC8445482; doi:10.1371/journal.pone.0257269)
Supplement: Attachment — Submitted filename: Response to Reviewers.docx [file pone.0257269.s001.docx]

Dear Dr. Komiyama and reviewers,

Thank you for your comments and questions regarding “The effects of cholesterol accumulation on Achilles tendon biomechanics: A cross-sectional study”. I thank you for your encouraging words surrounding the importance/interesting nature of this exploratory study.

Reviewer #1.

*“It is not clearly mentioned about cholesterol and apolipoprotein levels in the experimental but also in the control group.”*

I have collected clinical characteristics for the participants with FH from their yearly visit to the FH clinic (results closest to their data collection date), CG clinical characteristics were not able to be collected. However, each CG participant stated that they were healthy (to the best of their knowledge) and were not currently taking any form of medication that may confound our findings.

Added revision at line #224. “Clinical characteristics for participant’s with FH (CG not available) retrieval revealed a mean Dutch Lipid Clinic Network Score of 18±6 and a moderately controlled lipid profile (LDL: 3.5±1.6 mmol/L, total cholesterol: 5.4±1.6 mmol/L, apolipoprotein B:1.02±0.36 g/L).”

*“Did the patients were under hypolipidemic treatment or did they received such a treatment in the past? This is an important issue as treatment may affect considerably tendons properties.”*

All but one of the participants with FH were currently using lipid lowering medications to manage the disorder. This was expected as the more serious FH cases tend to present with xanthoma (adds an extra 6 points on to the Dutch lipid clinic network score, which explains why participants with FH had a mean score of 18). In the limitations, I have stated that it is not possible for this study to report correlations between severity of disorder or use of lipid lower and AT properties, due to the unknown size or severity of the AT TXT.

I have added a revision at line #221. “Each participant with FH was noted to be positive for TXT within the registry medical charts and all but one participant with FH were currently using one or more lipid lowering medications (mean usage 7.7±7.2 years).”

*“A minor comment is the rather limited number of subjects in order to draw any definite conclusions and thus should be added in the limitations.”*

Added a revision at line #394. “Lastly, sample size was relatively restricted due to the shortage of patients with AT TXT diagnosis within the BC FH Registry. The lack of TXT reporting within the registry is likely due prioritization of other clinical outcomes and under reporting of inconclusive physical examinations (potentially cases with diffuse thickening). Therefore, due to the small sample size, drawing conclusions based on the results of the study should be approached with caution.

Reviewer #2.

1. *“The authors should explain the impact of specific group sizes on observed differences.”*

Added revision at line #394. “Lastly, sample size was relatively restricted due to the shortage of patients with AT TXT diagnosis within the BC FH Registry. The lack of TXT reporting within the registry is likely due prioritization of other clinical outcomes and under reporting of inconclusive physical examinations (potentially cases with diffuse thickening). Therefore, due to the small sample size, drawing conclusions based on the results of the study should be approached with caution.”

2. “*The authors should comment if they observed any differences between the female and male participants.”*

Added revision at line #216. “MANOVA analysis was performed to determine if sex played a role in the effect of cholesterol accumulation on the primary outcomes”.

Added revision at line #284. “Females and males were equally affected by AT cholesterol accumulation in k50-100% (p=0.886, F=0.021 and η2=0.001), hysteresis (p=0.832, F=0.046, η2=0.002), peak strain (p=0.683, F=0.17, η2=0.006) and peak AT force (p=0.904, F=0.015, η2=0.001). Sex was a not predictor for each of the main outcomes (p>0.05).”

*3. “Although the authors explained that they could not measure the extent of accumulation of lipids in the tendons, they should present data on serum concentration of the lipids if available.”*

Added revision at line #224. “Clinical characteristics for participant’s with FH (CG not available) retrieval revealed a mean Dutch Lipid Clinic Network Score of 18±6 and a moderately controlled lipid profile (LDL: 3.5±1.6 mmol/L, total cholesterol: 5.4±1.6 mmol/L, apolipoprotein B:1.02±0.36 g/L).”

Reviewer #3.

Thank you for your comments, the manuscript (The effects of familial hypercholesterolemia on Achilles tendon biomechanics: a cross-sectional study [2018]) held within University of British Columbia’s online repository (cIRcle) is an unpublished master’s thesis. The purpose of cIRcle is to act an open access digital repository for unpublished material produced by students at the university. Thesis submission to cIRcle is a graduation requirement for every thesis-based student at the university.

Alterations were made to the current submission (compared to the thesis) to a) address the results/discussion in a more concise and appropriate way, making it suitable for peer reviewed publication, b) consider concerns/suggestions from co-authors that had not been a part of the master’s committee.

I look forward to hearing your feedback concerning the revisions and thank you for your time.

Kip Squier
